# Differential effects of amphetamine on ultrasonic vocalizations and locomotor activity in a rat model of endogenous depression

**DOI:** 10.1038/s41598-025-27298-x

**Published:** 2025-12-08

**Authors:** Anamarija Banjac, Kristian Elersič, Marko Živin, Maja Zorović

**Affiliations:** 1 Brain Research Lab, Institute of Pathophysiology, Faculty of Medicine, University of Ljubljana, Ljubljana, Slovenia; 2 Mind & Brain Lab, Department of Psychology, Faculty of Arts, University of Ljubljana, Ljubljana, Slovenia

**Keywords:** Wistar-Kyoto rat, Ultrasonic vocalizations, Anhedonia, Amphetamine, Morphine, Neuroscience, Physiology

## Abstract

The Wistar-Kyoto rat strain (WKY) is a model for treatment-resistant depression, exhibiting behaviors indicative of anhedonia. Although anhedonia is measured using the sucrose preference test (SPT), 50-kHz ultrasonic vocalizations (USVs) hold promise as a complementary assessment tool. This study aimed to detect whether 50-kHz USVs differ in the WKY rats compared to control Wistar (W) rats. First, WKY and W rats were compared for their basal 50-kHz calls. Then, rats were repeatedly treated with four administrations of either amphetamine (AMPH) or morphine (MORPH), and 50-kHz calls were recorded. In addition, the drug-evoked locomotor activity, sucrose consumption in the SPT, and the time spent in the drug- vs. saline-paired compartment in the conditioned place preference (CPP) test were collected. WKY rats emitted fewer basal calls and fewer calls following the first and the fourth AMPH administration. Contrary to controls, AMPH did not affect the number of calls in WKY rats following the first administration. WKY also displayed reduced AMPH-induced locomotor activity and sucrose consumption in the SPT. MORPH did not affect USVs or locomotion in either strain. The results highlight the benefits of 50-kHz calls as a complementary behavioral marker for assessing anhedonia in preclinical models of depression.

**Supplementary Information:**

The online version contains supplementary material available at 10.1038/s41598-025-27298-x.

## Introduction

The Wistar-Kyoto rat strain (WKY) is a robust and practical animal model for studying treatment-resistant depression. The strain demonstrates a range of endocrine^1,2^ and behavioral^3–6^ features that parallel those observed in patients suffering from depressive disorder. Behaviors indicative of anhedonia have also been described in WKY rats. Anhedonia is a core symptom of depression in humans, defined as a diminished interest in stimuli or diminished pleasure in response to stimuli that were previously perceived as rewarding^7^. WKY displays reduced sucrose consumption in a sucrose preference test (SPT)^3,8^, greater social avoidance during social interaction tests with novel males and females^9^, and WKY mothers show reduced caregiving response to their offspring compared to the control animals^10^. The strain also exhibits disrupted monoamine signaling, particularly dopamine which is characteristic to anhedonia in humans^11,12^.

Behavioral assessment of anhedonia is almost exclusively executed with the SPT. Still, the SPT protocol can introduce unwanted drives, like hunger or thirst, that potentially confound the results^13^. Therefore, additional experimental tools that enable the study of affective responses would make an important contribution to advancing the current understanding of anhedonia in WKY rats. In this manner, rat ultrasonic vocalizations (USVs) can be highly valuable. Rat USVs are broadly categorized into three groups: 40-kHz calls emitted by pups when separated from mothers, and calls emitted by juvenile and adult rats, at 22-kHz range in aversive situations, and 50-kHz range in appetitive situations^14,15^.

50-kHz calls are interpreted in several ways, namely as an expression of arousal and positive states, as a social function in activities like play, mating and social buffering, a motivational state, and even an expression of aggression^14–17^. The most notable hypothesis, however, regards the emission of 50-kHz calls as a tool for communication between rats, specifically to express arousal and a positive affective state^18^. The notion that 50-kHz calls are an expression of a positive affective state is supported by findings on the emission of 50-kHz calls prompted by the activation of dopamine receptors in the nucleus accumbens (NAc)^19,20^. As mentioned previously, dopamine has an evident role in processing rewarding stimuli. In addition, specific types of 50-kHz calls are associated with expressions of positive states. For example, a broad category of frequency-modulated (FM) 50-kHz calls has been observed when rats were in contact with various appetitive stimuli^18^. Likewise, exposure to positive stimuli in rats has sometimes been accompanied by a subtype of 50-kHz FM calls, known as “trills”, although they are occasionally linked to negative contexts as well^21,22^. Trills or the prevalence of trills over constant-frequency “flat” call subtypes, are therefore similarly being associated with positive emotional states^23,24^.

Many drugs that elicit a positive affective state in humans are linked to the emission of 50-kHz calls in rodents^25–30^. Accordingly, they present promising agents for evoking 50-kHz calls and indirectly assessing positive affective states in preclinical studies on anhedonia. Despite the limited literature, emerging evidence suggests that the WKY model exhibits alterations in the emission of 50-kHz calls. For example, when tested for basal activity in a novel cage WKY rats exhibit increased latency to the first basal call and emit fewer basal 50-kHz calls overall compared to the control Wistar (W) line^31^. Some studies on the WKY model report the predominance of certain call types over others; however, the results are overall inconclusive^31,32^.

This study aimed to detect whether 50-kHz calls in response to amphetamine (AMPH) and morphine (MORPH) differ in the WKY rat model compared to control W rats. AMPH and MORPH were chosen due to their differences in pharmacodynamics. Both drugs promote dopamine release at nerve terminals in the NAc^33^, which is linked to the emission of 50-kHz calls. However, AMPH acts mainly through dopamine transporter and monoamine oxidase inhibition at presynaptic terminals of dopaminergic neurons projecting from the ventral tegmental area^34^. On the other hand, morphine stimulates µ-opioid receptors at the GABAergic terminals of the ventral tegmental area, which in turn disinhibit dopaminergic neurons^35^.

The effects of AMPH on 50-kHz calls have been reported extensively across multiple strains. AMPH increases the emission of 50-kHz calls after the first administration and results in an even greater response after repeated treatment, indicating sensitization^19,20,28,29,35,36^. On the other hand, MORPH has produced mixed results. While it either suppresses or has no effect on the emission of 50-kHz calls after the first administration^27,37–39^, it has led to an increase in vocalizations following repeated treatment in some instances^25^ and no change in others^38^. In addition, rats treated with MORPH approached the playback of 50-kHz calls emitted from one arm of the radial maze more often compared to rats treated with vehicle^40^.

This study evaluated the acute effects of AMPH and MORPH on 50-kHz calls, as well as their sensitization after four administrations in WKY and control W rats. Sensitization of 50-kHz calls was assessed as it is believed to reflect modifications in the emotional state following repeated drug experience^16,35,41^. Additionally, basal 50-kHz calls and drug-evoked trill, FM and flat subtypes were assessed. We hypothesized that WKY rats would emit fewer basal calls and fewer AMPH- and MORPH-evoked calls compared to control W rats, after the first and fourth administration. WKY would also presumably exhibit a decreased prevalence of call subtypes compared to control W rats. Drug response was further assessed with drug-evoked locomotor activity and conditioned place preference (CPP). Finally, sucrose consumption/preference in the SPT, a behavioral marker of anhedonia, was measured before and after drug administration.

## Methods

### Animals

Male W (*n* = 36) and WKY (*n* = 36) rats (8 weeks old, Charles River Laboratories, Sulzfeld, Germany) were housed in polycarbonate cages (Ehret IV, Mahlberg, Germany, floor area 1825 cm²), three per cage (same line, different treatment groups), with sterilized bedding material (Lignocel ¾ and Rehofix, JRS, Rosenberg, Germany), cellulose towels and cardboard tunnels. Cages were maintained in a colony room at 22–24 °C and 35–60% relative humidity, under a 12:12 h light/dark cycle (lights on at 7:00 a.m.), with ad libitum access to autoclaved water and a maintenance rodent diet (1320 Altromin, Lage, Germany). Rats were handled in 6 sessions (⩽ 5 min. per session) over two weeks before the protocol, following a standardized handling procedure developed in our lab. All procedures were approved by the Administration for Food Safety, Veterinary Sector, and Plant Protection of the Republic of Slovenia (Reference Number U34401-16/2023/9). Animals were treated according to Directive 2010/63/EU of the European Union, the National Veterinary Institute Guide for the Care and Use of Laboratory Animals and ARRIVE guidelines. Every effort was made to minimize the suffering of the animals.

### Drugs

D-Amphetamine sulfate (Cat. No. A-5580, Merck, Darmstadt, Germany) and morphine hydrochloride (Cat. No. 506387, Fagron, Capelle a/d IJssel, Netherlands) were both administered subcutaneously at a dose of 1 mg/kg, with doses expressed as the salt weights. Both drugs were dissolved in sterile 0.9% saline (SAL) and administered to the hip region at a volume of 1 ml/kg. Doses were selected based on previous studies that showed an increase in AMPH-induced 50-kHz calls^24,42,43^, a change in 50-kHz calls after repeated MORPH treatment^25^, and the establishment of conditioned place preference using AMPH and MORPH at 1 mg/kg^45^. Rats were assigned to six groups of 12, based on the treatment (AMPH, MORPH, or SAL) and the strain (W or WKY).

### Study protocol

**Fig. 1:**
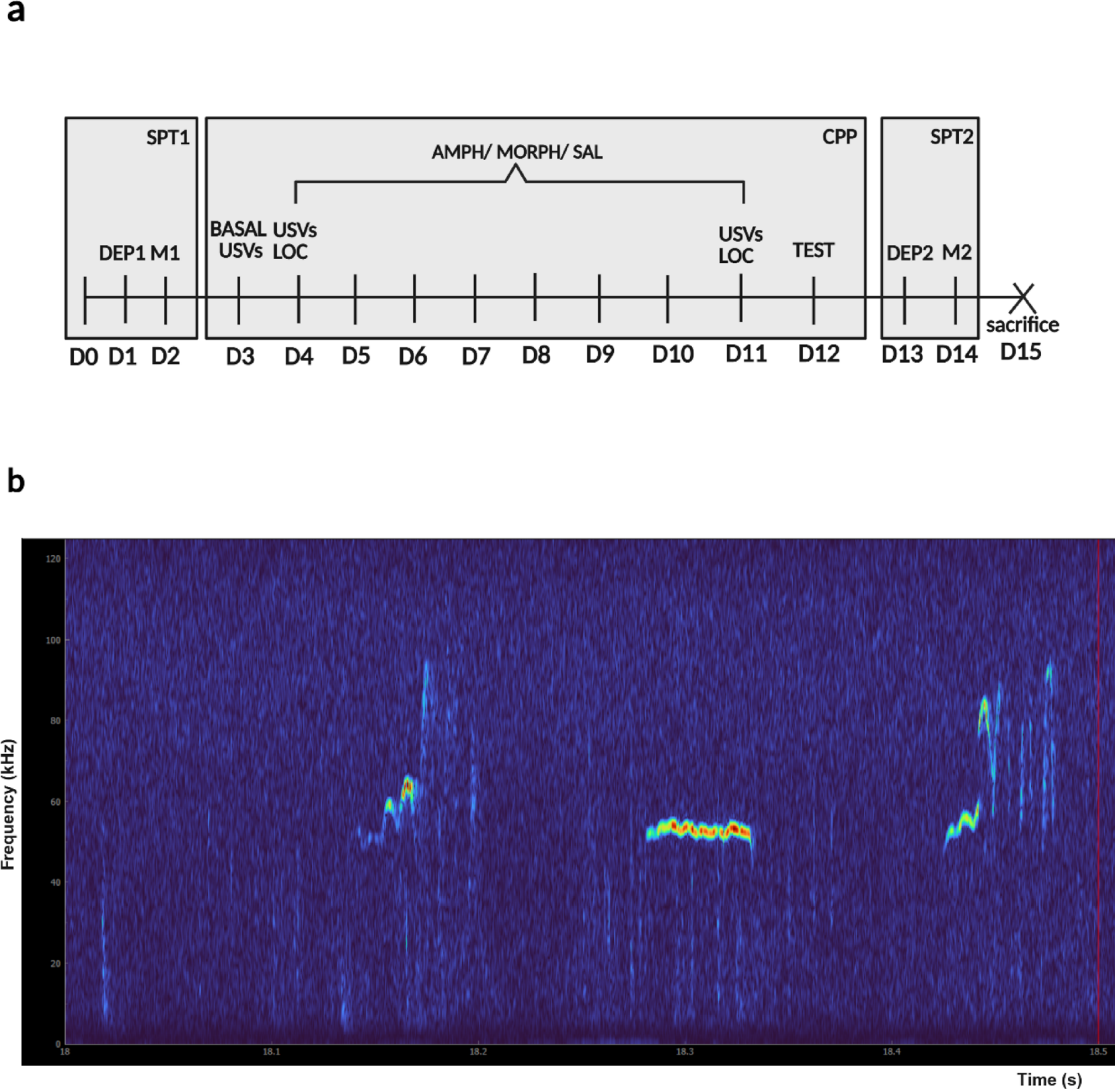
Study design. Panel a describes the study protocol. The experiment began when rats reached the age of 8 weeks. Initially, all rats underwent the first SPT (SPT1). Two days before the first measurement of sucrose consumption (day 0, D0), rats were habituated to two bottles in their home cage, which would later be used in the test: one containing fresh, autoclaved water and the other containing the sucrose solution. The following day (D1), they were deprived of water and food in their home cage. On the test day (D2), each rat was separated into individual cages, equipped only with water and sucrose solution bottles for a 12-h period. Subsequently (D3), rats were tested for their basal 50-kHz calls and habituated to the CPP arena for 15 min. Rats were conditioned (D4-D11) in the appropriate CPP compartments receiving either AMPH or MORPH (1 mg/kg s.c.) every other day with SAL injections in between. Their 50-kHz calls and locomotor activity were measured following the first and fourth administration. After 8 days (D12), rats were tested for CPP for 30 min. Lastly, rats were again deprived of water and food in their home cage (D13) and tested for sucrose consumption in equal conditions for 12 h (D14). The experiment ended with the rats’ sacrifice (D15). Panel b depicts a spectrogram with an example of 50-kHz calls. The spectrogram depicts examples for each type of assessed call. From left to right: FM call, flat, trill. CPP = conditioned place preference; TEST = CPP testing session; SPT = sucrose preference test; DEP1/DEP2 = food and water deprivation for 1 st and 2nd SPT test; M1/M2 = measurement of sucrose consumption; USVs = recording of 50-kHz ultrasonic vocalizations; LOC = test of locomotor activity; D0-D15 = days. Created in BioRender. Banjac, A. (2025) https://BioRender.com/xgl6n3p.

Testing began at 8:00 a.m., with rats being transported to the experimental room and weighed. Rats were always tested individually in the experimental room, equipped only with the strictly necessary electronics to minimize potential interferences with the recording equipment. All staff at the facility were kindly requested to minimize noise during testing hours. The protocol (Fig. 1a) was used to assess sucrose consumption in the SPT and the time spent in drug-paired vs. SAL-paired compartments in the CPP alongside acute drug-induced 50-kHz calls and locomotor activity.

### Conditioned place preference (CPP)

All measurements were conducted in the rectangular CPP arena, which was made entirely of plexiglass (120 cm in length, 80 cm in width, and 40 cm in height). The arena was divided into three equal compartments (40 cm in length, 80 cm in width, and 40 cm in height). The two side compartments featured detachable black walls made from plexiglass with patterns of either white circles or vertical white stripes. CPP was carried out according to the protocol adapted from Cunningham and colleagues^45^. Due to the high workload and a limited number of research staff, we decided to use a shorter version with 20-min. conditioning sessions^46–48^. The protocol spanned 10 consecutive days (D3-D12) with three phases: (1) Habituation; (2) Conditioning; and (3) Test.

1. *Habituation (D3)*: Rats were placed in the middle compartment and left to explore for 15 min. Their movement was monitored to assess preference for side compartments across all animals. No bias was detected.
2. *Conditioning (D4-D11)*: Rats were randomly assigned to one of three groups: AMPH, MORPH, or SAL. Over eight days, rats in AMPH and MORPH groups received alternating injections of the drug and SAL, commencing either with the drug or SAL (counterbalanced within the group). Immediately after each injection, rats were confined to the corresponding drug- or SAL-paired compartment for 20 min. The pairing of drug and SAL with side compartments and wall patterns was counterbalanced across groups. Rats in the SAL control group received only SAL injections in both compartments for eight days. Before the final drug administration (fourth dose), rats were placed in the drug-paired compartment for 10 min. to monitor anticipatory 50-kHz calls and locomotion (see Supplementary file 1).
3. *Test (D12)*: Rats underwent a 30-min. injection-free CPP test, starting in the middle compartment. Time spent in the drug- versus SAL-paired compartments was recorded as the measure of CPP.

Locomotor activity and 50-kHz calls were recorded simultaneously in the selected CPP compartment (40 cm x 80 cm x 40 cm) during conditioning sessions (except basal calls). The behavior was recorded under infrared (IR) illumination and dim red light (2 lx) using a GigE monochrome, IR-sensitive camera (Basler, Ahrensburg, Germany) centrally placed above the arena. Locomotion was tracked and analyzed via EthoVision XT 17.0 (Noldus, Wageningen, Netherlands). Locomotor activity was analyzed over a 10–20 min. time interval after administrations to capture the same timeframe as 50-kHz calls.

### Recording and acoustic analysis of ultrasonic vocalizations

Each rat was recorded individually. All recording sessions were performed under the dim red light. 50-kHz calls were measured on three occasions: basal calls, drug-evoked call emission, and anticipatory call emission. For basal calls, rats were injected with SAL (1 ml/kg) and placed in a clear polycarbonate cage equipped with fresh bedding and a lid. An Ultrasound condenser CM16/CMPA microphone (Avisoft Bioacoustics, Nordbahn, Germany) connected to an UltraSoundGate 116 H recording interface (Avisoft Bioacoustics, Nordbahn, Germany) was positioned 40 cm above the cage. For measurements of drug-evoked call emission, each rat was injected with one of three solutions and placed in a single compartment of the CPP arena for 20 min. The same recording equipment was used, but the microphone was attached to the ceiling and placed centrally, 172 cm above the floor of the compartment where the rat was located. The microphone was positioned further than usual because it was fixed parallel to the recording camera in a setup that enabled precise tracking of an animal with EthoVision. Such a setup for visual tracking requires an overview of the whole arena for all recordings. We successfully confirmed 50-kHz calls at this distance with additional preliminary testing conducted before the experiment.

The sampling rate was 250 kHz with 16-bit resolution. All audio files were analyzed for the second half of the 20-minute recordings. This time frame was chosen based on preliminary testing from our lab and previous reports that showed AMPH-induced calling is most pronounced within 10–20 min. time interval after administration^23,36^. Rats’ calls were recorded on one additional occasion, which was 10 min. before the last drug administration, under the same conditions as described previously (more details in Supplementary file 1).

Spectrograms were generated with DeepAudioSegmenter^49^. Spectrograms were generated with a short-time Fourier transform (STFT) length of 512 points and an overlap of 50% (Hann window, 100% frame size). An example of spectrogram with 50-kHz calls is shown in Fig. 1b. 50-kHz calls were manually detected and classified by a single individual (A.B.), blinded to the line and treatment conditions. 50-kHz calls were classified into the following four categories: trill, FM, flat, and other (call types not categorized into any of the first three groups). Trills, FM calls and flats were identified based on categories described by Wright and colleagues^23^. In addition, call types were labeled based on the following spectro-temporal criteria. Trill calls were defined as calls containing at least three frequency oscillations of 20 kHz or more, each oscillation lasting 15 ms or longer, appearing either sinusoidal or as repeated inverted-U patterns (Fig. 1b, right). Flat calls were characterized by frequency oscillations below 5 kHz and a minimum duration of 50 ms (Fig. 1b, middle). Of the remaining calls, those that exceeded a duration of 12 ms and included any kind of frequency oscillation larger than 5 kHz were categorized as FM calls (Fig. 1b, left). Given the labor-intensive nature of detection and classification, we measured the total call emission and the trill, FM and flat subtypes after the first and fourth drug administrations. This study aimed to examine 50-kHz calls. 22-kHz calls were not evaluated. Additionally, a negligible number of 22-kHz calls were observed during the recording sessions.

### Sucrose preference test (SPT)

Rats underwent SPT twice, at the start and at the end of the experiment, using an adapted protocol^50^. Each SPT consisted of 24 h of food and water deprivation in the home cage (DEP1/DEP2), followed by a 12-hour test session (M1/M2) (Fig. 1a). During testing, each rat was placed individually in a clear polycarbonate cage with fresh bedding and two bottles: one containing 250 ml of fresh, autoclaved water and the other containing 250 ml of 2% w/v sucrose (Cat. No. S0389, Sigma-Aldrich, Steinheim, Germany) mixed with fresh, autoclaved water. To control the side preference, the position of bottles was switched between animals in both tests.

Consumption was assessed by measuring the weight of the bottles before and after the session. An equipment malfunction resulted in missing data in three instances (*n* = 1 for SPT1; *n* = 2 for SPT2). In addition, three rats showed no sucrose preference, i.e. they consumed equal or less sucrose compared to water (two W rats (SAL and MORPH treatment group) and one WKY rat (SAL treatment)); all showed no preference on SPT2). These animals were included in the analysis in the same manner as other animals. Sucrose consumption was normalized by body weight (to account for weight differences between the lines). Before the first deprivation, all animals were habituated to the test bottles in their home cages for 24 h.

### Statistical analyses

Analyses and graphs were generated with R version 4.4.3^51^. The outcome measures included the number of 50-kHz calls and the distance moved during the 10–20 min. interval following the first and fourth administrations, sucrose consumption normalized by body weight, and the time spent in drug- and SAL-paired compartments during the CPP test. Normality and homoscedasticity were assessed using residual plots and Levene’s test.

Before analyses, 50-kHz call counts were logarithmically transformed to account for non-normality and heteroscedasticity. Differences between lines in treatment-dependent call emission and locomotion were assessed using between-subjects 2 × 3 (Line x Treatment group) ANOVAs. Differences within lines were assessed using separate within-subjects 2 × 3 ANOVAs (Administration x Treatment group). Post-hoc group differences were evaluated using either Welch’s or paired-sample t-tests, with Bonferroni correction for multiple comparisons. CPP and SPT results were analyzed using between-subjects 2 × 3 (Line x Treatment group) ANOVAs (with Type III ANOVA accounting for unequal group sizes in the second SPT) or Welch t-tests, all of which were Bonferroni-corrected. The *p* threshold for significance was set to 0.05.

Results are described with F-values, their accompanying degrees of freedom, *p*-values, and effect sizes expressed as omega squared mean values and their 95% confidence intervals. Results are also presented graphically as bar plots. For 50-kHz calls, plots were generated with a log-scaled y-axis (using ggplot’s *scale_y_log10()* function), with values on the y-axis reflecting non-transformed raw values. The height of bar plots shows non-transformed means, and whiskers depict the standard error of the mean (SEM). Dot plots in each group depict values for each individual. Descriptive statistics are reported in Supplementary file 1 and reflect log-transformed values when applicable.

Trill, FM and flat call types were binarized (0 = no emission of respective call type, 1 = emitted respective call type) due to the high count of null observations after treatment with MORPH and SAL. First, calls from both the first and fourth administrations were jointly analyzed. Separate binary logistic regression models were fitted to assess the association between the probability of emission of trill, FM or flat calls and two factors: line (ref = W) and drug treatment (ref = SAL treatment). Next, the data was split for the first and fourth administration. Again, separate logistic regression models were fitted to examine the association between the probability of emission of trill, FM or flat calls and factors, including the line (ref = W) and drug treatment (ref = SAL). Adjusted odds ratios, McFadden’s R^2,^ and Akaike Information Criterion (AIC) are reported for significant models.

## Results

### Basal 50-kHz calls

**Fig. 2:**
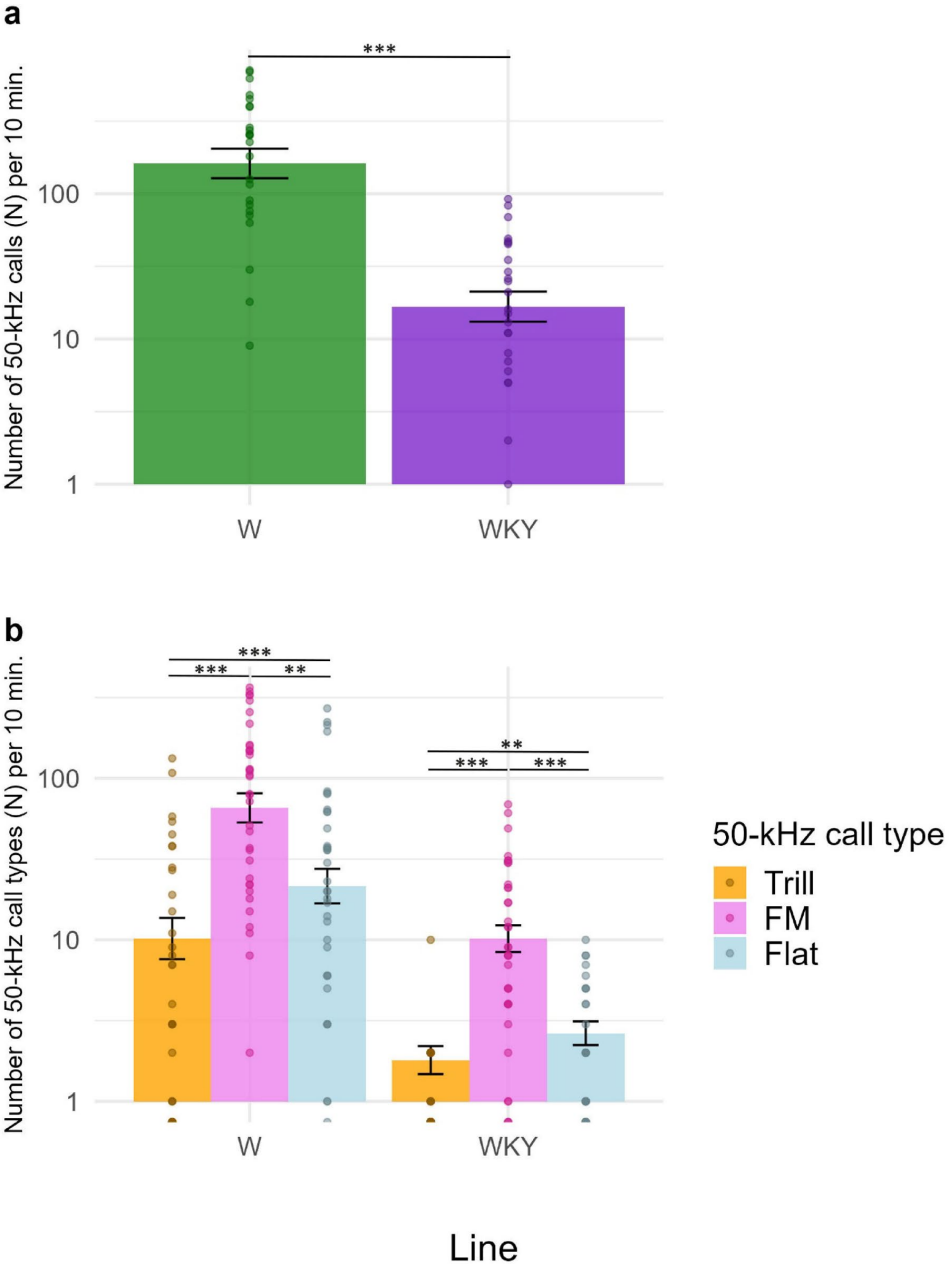
*Basal 50-kHz calls in W and WKY rats.* Panel a describes the number of basal 50-kHz calls in W and WKY rats. Panel b describes the number of trill, FM and flat 50-kHz call types in the W and WKY line. Basal 50-kHz calls were recorded for 10 min. Following the SAL injection. *n* = 36 per group. The data are presented as the mean ± SEM. The dots represent individual animals. W = Wistar rats; WKY = Wistar-Kyoto rats; FM = frequency-modulated 50-kHz calls. ** *p* < 0.01; *** *p* < 0.001.

The results are displayed in Fig. 2. Welch’s two-sample t-test showed that WKY rats emitted significantly less 50-kHz calls compared to W rats (Fig. 2a, t = 7.78; df = 69.94; *p* < 0.0001, two-tailed). Two-way within-subjects ANOVA revealed a significant interaction between line and 50-kHz call type (*F*(2,140) = 3.71; *p* = 0.0269; ⍵^2^ = 0.07 with 95% CI [0.01, 0.16]). Both lines emitted the highest number of FM 50-kHz call types, followed by flats and the lowest number of trills (Fig. 2b).

### Drug-evoked 50-kHz calls and locomotion

**Fig. 3:**
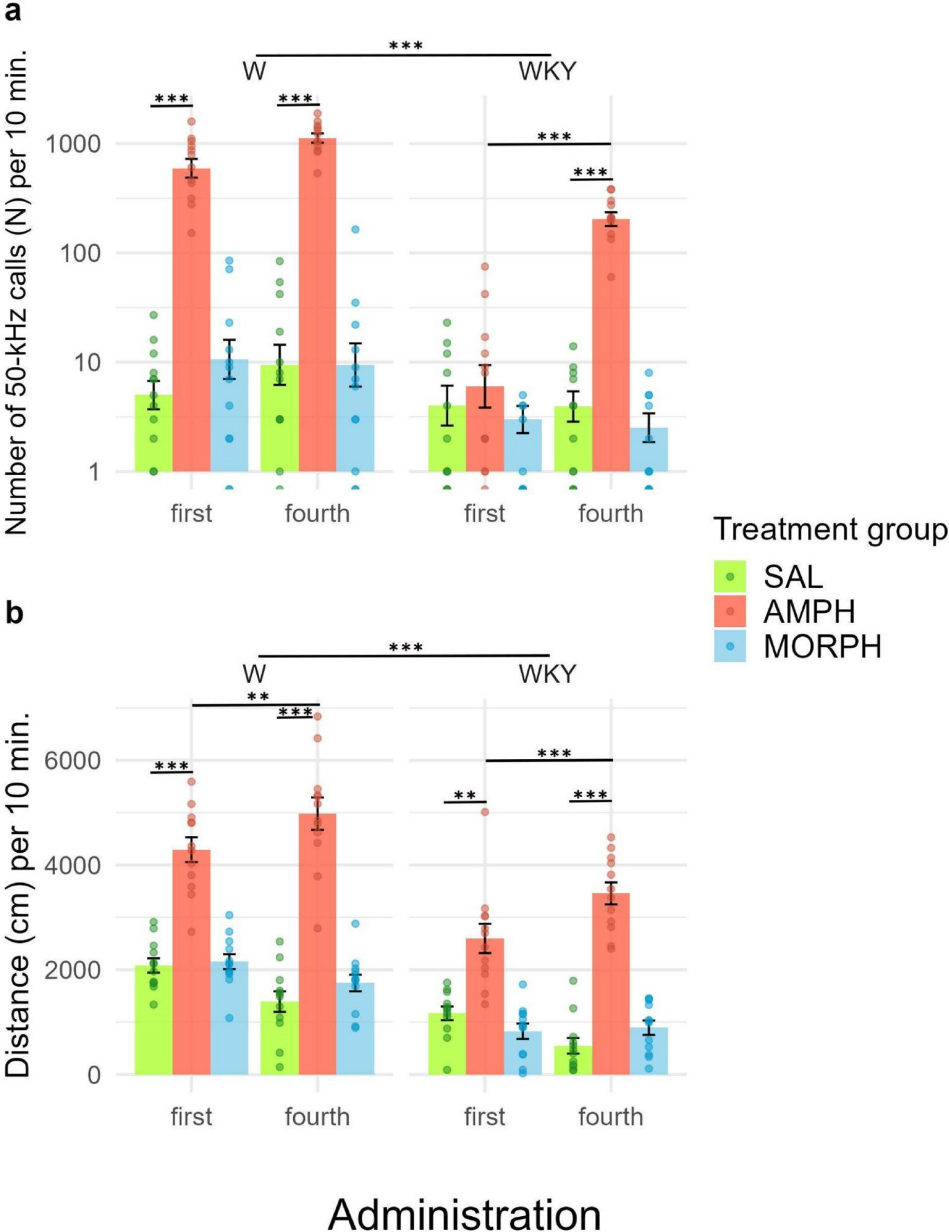
50-kHz calls and locomotion in W and WKY after treatment with AMPH or MORPH. Panel a describes the number of 50-kHz calls after the first and fourth administration of either SAL, AMPH, or MORPH in W and WKY rats. Panel b describes the distance traveled (cm) after the first and fourth administration of either SAL, AMPH, or MORPH in W and WKY rats. Emission of 50-kHz calls and locomotor activity were recorded for 20 min. The present results show 10–20 min. time interval after administration. *n* = 12 per group. The data are presented as the mean ± SEM. Dots represent individual animals. W = Wistar rats; WKY = Wistar-Kyoto rats; SAL = saline; AMPH = amphetamine; MORPH = morphine. * *p* < 0.05; ** *p* < 0.01; *** *p* < 0.001.

Results are displayed in Fig. 3. Two-way between-subjects ANOVA revealed a significant main effect of line on the number of 50-kHz calls (*F*(1,138) = 65.19; *p* < 0.0001; ⍵^2^ = 0.31 with 95% CI [0.18, 0.43]). WKY emitted a significantly lower number of calls than W (Fig. 3a, *p* < 0.0001). A significant interaction between line and treatment group was also revealed (F(2, 138) = 10.70; *p* < 0.0001; ⍵^2^ = 0.12 with 95% CI [0.03, 0.23]). In both lines, AMPH-treated animals emitted more calls than SAL-treated animals (W: *p* < 0.0001; WKY: *p* < 0.0001). In addition, WKY emitted significantly less 50-kHz calls than W in every treatment group (SAL: *p* = 0.0249; MORPH: *p* = 0.00019; AMPH: *p* < 0.0001).

Two-way within-subjects ANOVAs revealed a significant interaction between treatment group and administration only in WKY rats (*F*(2,60) = 8.52; *p* = 0.00055; ⍵^2^ = 0.17 with 95% CI [0.02, 0.35]). The number of 50-kHz calls in WKY rats increased from first to fourth AMPH administration (*p* < 0.0001). The number of 50-kHz calls in W rats did not differ significantly between the first and fourth AMPH administrations. MORPH did not affect call emission in either line.

For locomotor activity, a two-way between-subjects ANOVA showed a significant main effect of line on the traveled distance (*F*(1,138) = 95.95; *p* < 0.0001; ⍵^2^ = 0.40 with 95% CI [0.27, 0.52]). WKY rats moved significantly less than W across all conditions (Fig. 3b, *p* < 0.0001). The same ANOVA test revealed a significant main effect of treatment (*F*(2,138) = 184.65; *p* < 0.0001; ⍵^2^ = 0.72 with 95% CI [0.63, 0.79]). AMPH-treated animals traveled a larger distance compared to SAL-treated (*p* < 0.0001) and MORPH-treated animals (*p* < 0.0001). A trend towards significant interaction between the line and treatment group was also observed (F(2, 138) = 3.17; *p* = 0.0451; ⍵^2^ = 0.03 with 95% CI [0.00, 0.10]). In both lines, AMPH-treated animals moved significantly more than SAL-treated animals (W: *p* < 0.0001; WKY: *p* < 0.0001).

Two-way within subject ANOVAs showed a significant interaction between treatment group and administration within both lines (W: *F*(2,33) = 12.94; *p* < 0.0001; ⍵^2^ = 0.25 with 95% CI [0.02, 0.48]; WKY: *F*(2,33) = 13.04; *p* < 0.0001; ⍵^2^ = 0.25 with 95% CI [0.02, 0.48]). Traveled distance increased from first to fourth AMPH administration in both lines (W: *p* = 0.006; WKY: *p* = 0.00081). MORPH did not affect locomotor activity in either line.

### Probability of trill, FM and flat call emission

**Fig. 4:**
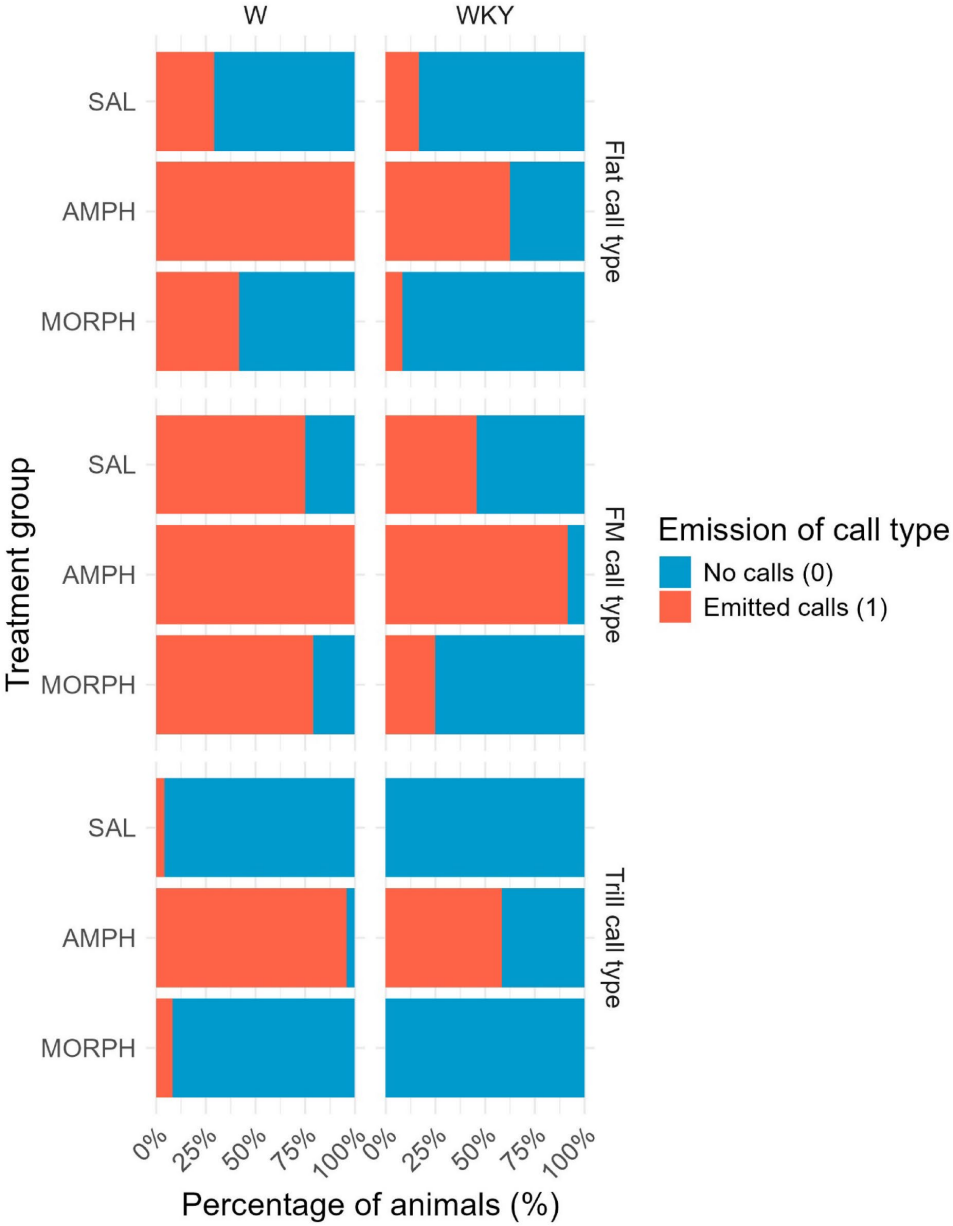
depicts the descriptive data on the percentage of animals that emitted trill, FM and flat call types in each treatment group. The number of trills, FM calls and flats in each experimental group is described in Supplementary file 1.

Figure 4. *The percentage of animals in each line that emitted at least one respective call type when treated with SAL*,* AMPH*,* or MORPH.* 50-kHz calls were classified into the following four categories: trill, FM, flat, and other (all other call types). Trill, FM and flat call types were analyzed. Call types were binarized (0 = no emission of respective call type, 1 = emitted respective call type) due to the high count of null observations. *n* = 24 per group. W = Wistar rats; WKY = Wistar-Kyoto rats; SAL = saline; AMPH = amphetamine; MORPH = morphine; FM = frequency-modulated 50-kHz calls.

The regression model examining the association of line and treatment showed that only treatment was a significant predictor (ref = SAL) for the probability of emitting a trill call. The odds of rats emitting trills were 529 times higher for treatment with AMPH than for treatment with SAL (AOR = 529, McFadden R^2^ = 0.63; AIC = 75.00). No significant predictor was found for the probability of emitting a flat call. For the probability of emitting FM calls, treatment was not a significant predictor, but the line was. The odds of WKY rats emitting FM calls are 72% lower than for W rats (AOR = 0.28; McFadden R^2^ = 0.29; AIC = 137.42). The predictors for the model are presented in Table 1.

**Table 1:** Overall probability of *trill and FM call* emission vs. line and drug treatment.

Probability of trill call emission vs. line and drug treatment			
Characteristic	AOR	95% CI	*p*-value
Intercept	0.04	0.00, 0.21	0.002
Line			
*W*	–	–	–
*WKY*	0.00	–	0.99
Treatment			< 0.001
*SAL*	–	–	–
*AMPH*	529	50.73, 19 841.45	< 0.001
*MORPH*	2.09	0.19, 46.90	0.558
Line x Treatment			
*W x SAL*	–	–	–
*WKY x AMPH*	831970.55	0.00, 6.42 × 10^18^	0.99
*WKY x MORPH*	0.48	0.00, 3.91 × 10^42^	0.99

Probability of *FM call* emission vs. line and drug treatment			
Characteristic	AOR	95% CI	*p*-value
Intercept	3.00	1.26, 8.28	0.020
Line			< 0.05
*W*	–	–	–
*WKY*	0.28	0.08, 0.93	0.043
Treatment			
*SAL*	–	–	–
*AMPH*	38549597.63	4.11 × 10^− 27^, 4.08 × 10^218^	0.990
*MORPH*	1.27	0.33, 5.10	0.732
Line x Treatment			
*W x SAL*	–	–	–
*WKY x AMPH*	0.00	0.00, 8.40 × 10^16^	0.991
*WKY x MORPH*	0.31	0.05, 1.90	0.209

Adjusted odds ratios with 95% confidence intervals and p-values for the probability of trill and FM call emission vs. line and treatment. AOR = adjusted odds-ratio, CI = confidence interval. *N* = 144.

Separate models for the first and fourth administration showed a significant association between the probability of emitting trill and treatment only after the fourth administration. The probability of rats emitting trill calls after the fourth administration was 121 times higher for treatment with AMPH than for treatment with SAL (AOR = 121; McFadden R² = 0.78; AIC = 32.65). No significant predictor was found for the probability of emitting flat or FM calls following the first or fourth administration. The predictors for the model after the fourth administration are presented in Table 2.

**Table 2:** Probability of *trill call* emission after fourth drug administration vs. line and drug treatment.

Characteristic	AOR	95% CI	*p*-value
Intercept	0.09	0.01, 0.47	0.002
Line			
*W*	–	–	–
*WKY*	0.00	–	0.997
Treatment			< 0.001
*SAL*	–	–	–
*AMPH*	121	10.52, 4714.53	0.0011
*MORPH*	1	0.04, 27.48	0.999
Line x Treatment			
*W x SAL*	–	–	–
*WKY x AMPH*	6.57 × 10^15^	0.00, 4.23 × 10^18^	0.995
*WKY x MORPH*	1	0.00, 1.16 × 10^44^	0.99

Adjusted odds ratios with 95% confidence intervals and p-values for the probability of trill call emission vs. line and treatment. AOR = adjusted odds-ratio, CI = confidence interval. *N* = 72.

### Conditioned place preference

**Fig. 5:**
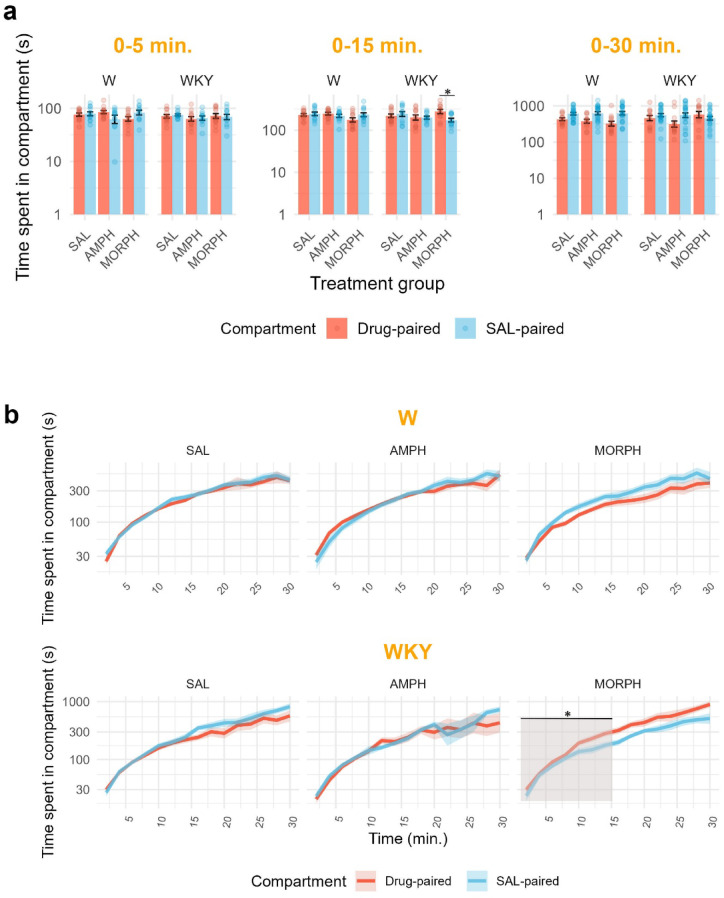
*Time spent (s) in drug-paired vs. SAL-paired compartments in W and WKY rats after treatment with AMPH*,* MORPH*,* and SAL.* After 8 days of conditioning sessions, rats were tested in the CPP arena for 30 min. Panel a depicts the difference between time spent in the drug-associated vs. SAL-associated compartment after 5 min., 15 min. and 30 min. Dots represent individual animals. Panel b depicts change over time in every group. Inferential analyses were conducted after animals were in the CPP arena after 5 min., 15 min. and 30 min. *n* = 12 per group. The data are presented as the mean ± SEM (bar and errorbars in Panel a; lines and shaded area around in Panel b). W = Wistar rats; WKY = Wistar-Kyoto rats; SAL = saline; AMPH = amphetamine; MORPH = morphine.

The results are depicted in Fig. 5. To control family-wise error rate, time spent in side compartments was tested after 5 min., after 15 min. and after the whole 30 min. rats spent in the arena (Fig. 5a). Change over time is depicted in Fig. 5b. Separate two-way ANOVAs for each line revealed a significant interaction only when first 15 min. were tested (*F*(2,64) = 3.34; *p* = 0.0415; ⍵^2^ = 0.06 with 95% CI [0.00, 0.19]). WKY rats spent significantly more time in MORPH-associated than SAL-associated compartment.

### Sucrose preference

**Fig. 6:**
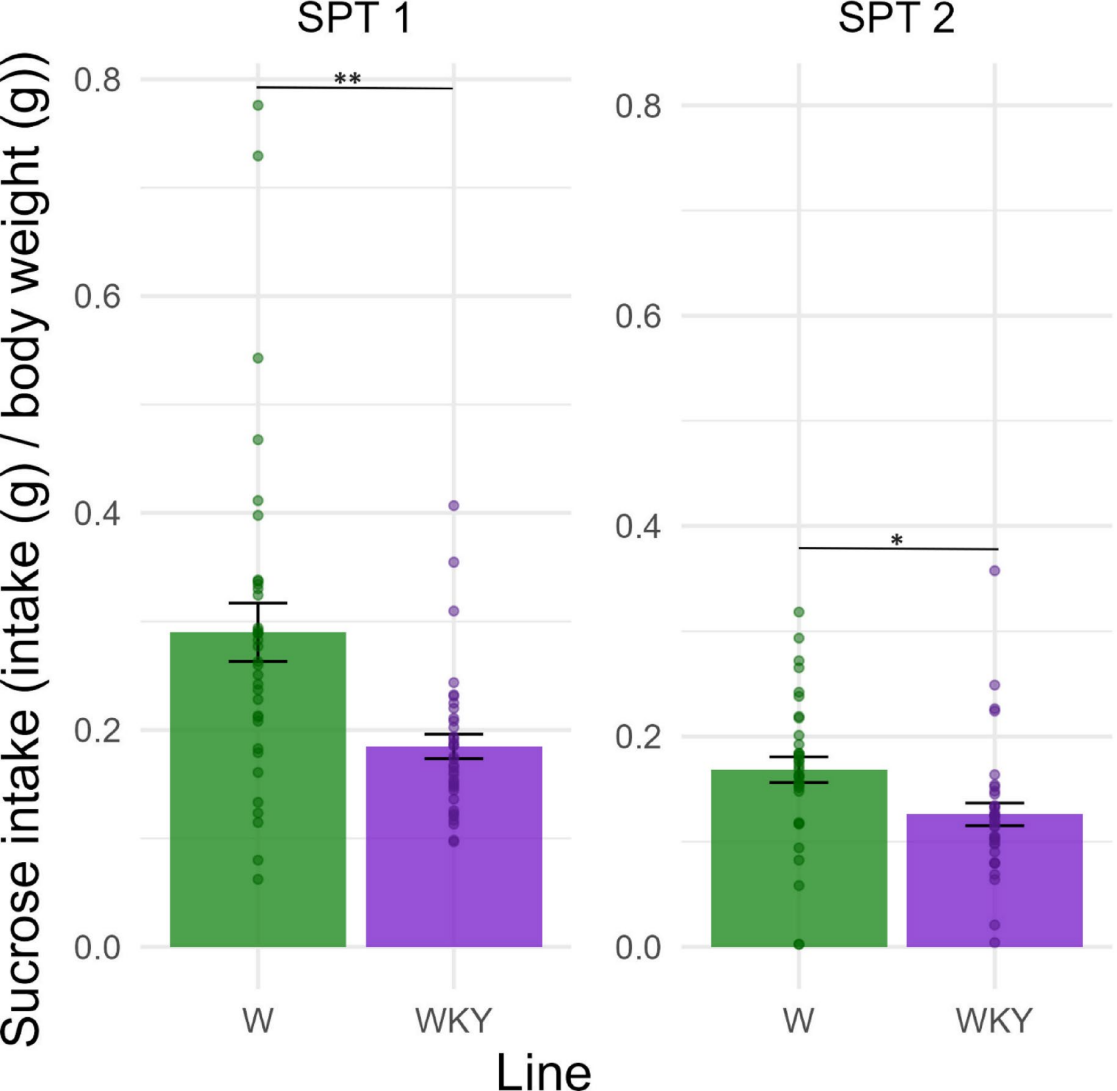
Sucrose intake (sucrose solution drank (g)/body weight (g)) in W and WKY rats before (SPT1) and after (SPT2) drug treatment. Rats were subjected to a 12-h test session for each SPT. All bottles were weighed before and after the session. The amount of water and sucrose drunk was taken as a measure of water and sucrose consumption. A malfunction in the equipment resulted in missing data in three instances (*n* = 1 for SPT1; *n* = 2 for SPT2). Sucrose consumption was normalized by body weight (to account for weight differences between lines). *n* = 33–36 per group. The data are presented as the mean ± SEM. Dots represent individual animals. SPT = sucrose preference test; W = Wistar rats; WKY = Wistar-Kyoto rats. ** *p* < 0.01.

Two-sample two-tailed t-test for SPT1 and two-way between subjects ANOVA for SPT2 revealed a significant difference in sucrose consumption between the lines after the first and the second SPT (SPT1: *t* = 3.67, df = 45.80, *p* = 0.0006; SPT2: *F*(1,63) = 6.91; *p* = 0.0107; ⍵^2^ = 0.08 with 95% CI [0.00, 0.24]). WKY rats consumed less sucrose solution compared to W rats in both tests. Drug treatment did not influence sucrose intake in the second SPT. Results are displayed in Fig. 6.

## Discussion

This study aimed to assess whether 50-kHz calls can serve as an additional measure of anhedonia in WKY rats by detecting the difference in the emission following the neutral and positive stimuli. WKY and control W rats were compared for their basal 50-kHz calls and following repeated AMPH and MORPH treatment. We hypothesized that WKY rats would emit fewer basal calls and fewer AMPH- and MORPH-evoked calls compared to control W rats after the first and fourth administrations. WKY would also presumably exhibit a decreased prevalence of call types compared to control W rats. In addition, drug response was assessed with drug-evoked locomotor activity and CPP, and anhedonia was confirmed with the sucrose consumption in the SPT.

In this study, WKY rats emitted fewer 50-kHz calls than W rats across all conditions. First, WKY rats emitted fewer basal calls, consistent with previous findings^30^. While we could not find any reports on AMPH-induced 50-kHz calls in WKY rats, some findings on other depression models report a reduced number of 50-kHz calls^43,52^. In this study, WKY rats emitted fewer 50-kHz calls compared to W rats following both the first and fourth AMPH administrations.

Additionally, the first AMPH administration did not increase 50-kHz calls in WKY rats; the increase was observed only after the fourth administration. This suggests that 50-kHz calls were nonetheless sensitized in WKY rats. In contrast, control W rats showed elevated 50-kHz calls after the first AMPH administration. The increase in calling was observed from the first to the fourth administration, though not at a significant level, suggesting a lack of sensitization, contrary to previous findings^19,20,23,28,29,35^. The absence of significant effect in W rats may be attributed to the subcutaneous route of administration. Indeed, subcutaneous administration is linked to slower absorption^53^, which could mean that W rats did not show significant sensitization to AMPH in this study simply because not enough time passed for AMPH to show full effects. That said, sensitization of 50-kHz calls following repeated subcutaneous AMPH administration is reported^54^, albeit at a higher dose (2 mg/kg) and up to 90 min. following the administration.

Nevertheless, this difference indicates that the same AMPH dose that was able to induce a significant sensitization in WKY rats was not as effective in W rats, despite WKY rats emitting fewer 50-kHz calls following both the first and fourth AMPH administrations. Accumulative mechanisms behind behavioral sensitization highly resemble long-term brain plasticity changes observed in human addiction^55,56^. Sensitization has been extensively used in preclinical research^35,56,57^ and is considered an indicator of addiction. Some findings suggest that WKY rats have a higher susceptibility to addictive behaviors related to alcohol and sugar consumption^58–60^. Still, this increased susceptibility was not found for cocaine, nicotine, or sucrose pellet self-administration^61,62^. Therefore, it remains unclear if WKY rats are generally more prone to addiction compared to other strains.

Reconsidering the previous position, alternative explanation for the change in AMPH-induced 50-kHz calls in WKY rats does not involve sensitization at all. During the first AMPH administration, WKY rats had been possibly introduced to novel, stressful environment that blunted the call emission. Still, locomotor effects were visible after the first AMPH administration and locomotor sensitization also occurred, so we can speculate that AMPH affected WKY rats from the start. Further, if the stress response prompted by novelty could blunt the expression of 50-kHz calls, that is still valuable information about strain-specific characteristics not observed in W rats.

Furthermore, the call profiles of both rat lines during basal measurements were characterized by a predominance of FM calls, followed by flats, with trills exhibiting the lowest occurrence. Interestingly, the probability of emitting FM calls was dependent on the line, with WKY rats being less likely to emit FM calls irrespective of treatment they received. This may point to inherent variations between the strains. FM calls are associated with expressions of positive states^18^, and WKY rats demonstrate attenuated hedonic responsiveness on other behavioral measures^3,9,10^. Reduced tendency to emit FM calls could be another behavioral indication of blunted hedonic state. AMPH treatment in this study increased the probability of emitting trill calls irrespective of the line. Other findings with control animals indicate an increase in trill calls or predominance of trills over flats following the repeated AMPH treatment^23,24^. Trill calls in rats are sometimes linked to positive states^23^. We assumed that we would observe a difference between lines in the emission trill calls in response to drugs. Such difference was not observed. Our findings demonstrate that a broader category of FM calls may indicate more about strain differences than narrowly defined trill calls. Having said that, the data on the emission of call types in WKY rats is scarce and inconsistent^30,31^. Therefore, future studies that measure 50-kHz call types in various positive situations could clarify if and how they differ from the control lines.

MORPH did not produce any effects on 50-kHz calls in this study. Although single MORPH administration either suppresses or does not affect the emission of 50-kHz calls^27,37–39^, repeated administration results in an increase in calling^25^. To our knowledge, no studies have examined the effects of MORPH on 50-kHz calls in WKY rats or other depression models.

Locomotor activity in WKY rats was reduced across all conditions, with distinct responses seen only following the AMPH administration. Previous studies have also reported decreased baseline locomotion^63^, attenuated locomotor responses to acute AMPH challenge^64^, and reduced locomotor responses following the chronic combined treatment of alcohol and AMPH^65^ in WKY rats. Similar locomotor deficits in response to AMPH have been reported in other depression models^66–68^. Both WKY and control W rats showed locomotor sensitization with repeated AMPH exposure.

Remarkably, WKY rats demonstrated an elevated locomotor response to the first AMPH administration, contrary to 50-kHz calls. This discrepancy was not observed in W rats. Divergence in locomotor responses and USVs is a well-documented phenomenon^16,28,43^, but it has not been reported in the WKY model yet. Some suggest that reduced 50-kHz calls indicate a lower affective response to stimulants, independent of dopaminergic activation in the mesolimbic system, as demonstrated by locomotor sensitization^41^. Still, the relation between those measures remains unclear. Sensitization of 50-kHz calls may also be attributed to underlying processes in sensitization, like synthesis of specific proteins, that require time to develop a fitting response^56^. Additionally, differential vocal and locomotor response potentially stems from different contributions of dopamine receptor subtypes, with dopamine D1 receptors altering USVs to a greater extent than D2 receptors^69^. WKY rats display reduced dopamine D1 receptor binding^11^ and altered distribution of D1, D2, D3, and dopamine transporter sites^11,12,70^ in the NAc, suggesting that the observed divergence in this strain may be related to the neurochemical differences in D1 receptors. Future studies in WKY rats that examine vocal responses after D1 and D2 antagonism could further clarify this relation.

Furthermore, WKY rats in this study exhibited reduced sucrose consumption in both SPT tests, consistent with previous reports^3,62,71^, and confirming anhedonia in the strain. Neither AMPH nor MORPH affected sucrose intake. This study found that the WKY rat line, which displays a diminished response to sucrose, a standard reward-like stimulus, also exhibits a reduced USV response to AMPH, a presumably reward-like stimulus as well. 50-kHz USV calls can therefore be implemented as a complementary, non-invasive measure of anhedonia, which can be easily incorporated into behavioral protocols.

Drug-dependent behavior was not observed in the CPP paradigm. We tested CPP effects in three time frames: after 5 min., after 15 min. and for the whole 30 min. rats resided in the arena. The only effect observed was a significant preference for MORPH in WKY rats after 15 min., but this result should be approached with caution. With *p*-value of 0.04, small effect size, and other time frames showing no effect, this result is likely a product of family-wise error rather than a robust effect. Nevertheless, a preference for the MORPH-associated compartment is described in other studies for the dose of 1 mg/kg in control rats^44,72–74^ and the dose of 1.25 mg/kg in WKY rats^75^. A preference for AMPH-associated compartment is also extensively reported in other studies, using the exact dosage, the same or fewer training sessions as in this study, and a subcutaneous route of administration^44,76–78^. WKY rats were previously found to develop CPP for a similar compound, cocaine, albeit at higher doses and with more training sessions^79^. We were careful to adhere to established protocols^45,80^ and employ an unbiased experimental design^45,74,80^. It is possible, however, that more, and especially longer training sessions are necessary for the emergence of AMPH CPP, since in all previously mentioned protocols, each training session lasted at least 30 min. For MORPH, several studies report on the emergence of CPP with higher doses^75,81–83^. We decided on this MORPH dose because it was shown to induce CPP in some cases and elevate 50-kHz calls after repeated exposure^25^.

In the end, we acknowledge several limitations of this study. One possible reason for the absence of drug-dependent behavior in the CPP test is that anticipatory 50-kHz calls were recorded 10 min. before the final conditioning session in the drug-associated compartment (Supplementary file 1). This recording may have acted as an extinction session, disrupting the association between the drug and the drug-associated compartment. However, a complete extinction of drug-dependent behavior in the CPP paradigm typically requires 4 to 8 extinction sessions^84–86^. It is more likely that the association was not formed in the first place in this study, hence the lack of effect in the CPP test. Another potential reason for the lack of the CPP effect could be the dim red light used during all sessions, which may have prevented rats from seeing the patterns on the walls. Although there is much debate on this topic, the latest findings indicate that rats should indeed be able to see and discriminate stimuli under the red light^87,88^. Moreover, the emergence of CPP effect could be hindered by the setup of the arena itself. In the post-conditioning test session, rats spent considerable time in the middle compartment. The middle compartment in this setup is larger than in a regular CPP arena, so the drive for the exploration could have had overpowered the conditioned drug-place associations.

Next, this study used exclusively male subjects. Clinical research highlights pronounced sex differences in the neurobiology, etiology, symptomatology, and treatment response of depression^89–91^. Previous work has shown that female WKY rats do not exhibit the same anhedonic phenotype in the SPT as males^3^; however, 50-kHz calls have not been systematically investigated in WKY females. Given the known sex differences in laryngeal muscle and vocalization patterns in other strains^92^, future studies should include female rats to enhance the translational relevance of the WKY model.

Furthermore, call features like mean peak frequencies and call durations were not analyzed in this study. While different acoustic features of 50-kHz calls could be associated with differences in conveying emotional arousal, the sound frequency band remains the most informative and critical variable^18,93^. Based on that notion and limitations in extracting the call feature data from the program used for detection and classification of 50-kHz calls, this data was not analyzed.

Finally, caution is warranted when 50-kHz calls are interpreted exclusively as expressions of positive affective state. Although this hypothesis is well-supported, and 50-kHz calls are related to positive states^18–20,23,24^, these calls also occur in other contexts, like aggressive encounters between rats^17^. Moreover, rats can suppress 50-kHz calls even when they are in an intense positive state. In an experiment employing a “hide-and-seek” game between experimenters and rats, a game designed to evoke high positive arousal, rats could reduce their 50-kHz calling when hiding from experimenters^94^. This finding is related to the notion that the change in 50-kHz calls and FM call types can also convey information about affective memories^95,96^. Based on that, attenuated call emission of WKY rats in this study is possibly not just attributed to the expression of a positive state at given moment, but also memories of affective states in previous situations. Therefore, interpreting a reduced number of 50-kHz calls as an indication of a lower positive state should be done with caution.

## Conclusion

This study aimed to detect whether 50-kHz calls differ in the WKY rat model compared to control W rats. This study is the first to investigate 50-kHz call responses in the WKY model of depression following exposure to AMPH and MORPH, in addition to complementary behavioral measures, including locomotor activity, sucrose consumption in the SPT, and time spent in the drug-paired compartment in the CPP. WKY rats emitted fewer 50-kHz calls in all conditions, exhibited reduced locomotor activity, and consumed less sucrose in the SPT. Notably, 50-kHz calls in WKY rats diverged from their locomotor activity after the first AMPH administration. In contrast to 50-kHz calls, locomotor activity was elevated compared to the SAL treatment. This divergence suggests that 50-kHz call responses and locomotor activity may be relatively separate processes in WKY rats. By comparing 50-kHz calls with other behavioral measures, our results support 50-kHz calls as a complementary measure of anhedonia in WKY rats.

## Supplementary Information

Below is the link to the electronic supplementary material.

Supplementary Material 1

## Data Availability

The datasets generated and/or analyzed during the current study are available with the following OSF link: https://osf.io/ekxm2/?view_only=fae9f753b43243f886693609f648b22d.
