# Supplementary material for: Differential effects of amphetamine on ultrasonic vocalizations and locomotor activity in a rat model of endogenous depression
Source: Sci Rep. 2025 Dec 8;15:43328. doi: 10.1038/s41598-025-27298-x (PMC12686103; doi:10.1038/s41598-025-27298-x)
Supplement: Supplementary file 1 — Supplementary Material 1 [file 41598_2025_27298_MOESM1_ESM.pdf]

SUPPLEMENTARY FILE 1

50-kHZ CALLS: DESCRIPTIVE DATA

Below, we present a table (Supplementary table S1) with descriptive data for significant differences between groups tested for the number of 50-kHz calls. All data is log-transformed.

Table S1

			M	*SD*
	Context-free basal 50-kHz calls			
W	*overall*		4.92	1.08
WKY	*overall*		2.96	1.05
W	*trills*		1.61	1.63
	FM calls		4.18	1.25
	flats		2.98	1.53
WKY	*trills*		0.18	0.45
	FM calls		2.13	1.23
	flats		0.62	0.80
	50-kHz calls in response to all treatments			
W	*overall*		3.49	2.58
WKY	*overall*		1.68	1.95
	50-kHz calls in response to AMPH administration			
W	AMPH	*overall*	6.71	0.62
	SAL	*overall*	1.83	1.27
	AMPH	*1st*	6.39	0.68
		4th	7.02	0.34
WKY	AMPH	*overall*	3.48	2.17

	SAL	*overall*	1.03	1.12
	AMPH	*1st*	1.64	1.51
		4th	5.32	0.50

Means and standard deviations for all groups with significant differences in 50-kHz calls. M = mean, SD = standard deviation, W = Wistar line, WKY = Wistar-Kyoto line, AMPH = amphetamine, SAL = saline.

LOCOMOTION: DESCRIPTIVE DATA

Supplementary table S2 presents descriptive data on significant differences between groups tested for locomotion, expressed in terms of distance traveled. All data is presented non-transformed and expressed in centimeters.

Table S2

			M	*SD*
	Traveled distance in response to all treatments			
W		*overall*	2776. 21	1527.92
WKY		*overall*	1583.13	1236.61
	Traveled distance in response to AMPH administration			
W	AMPH	*overall*	4638.15	998.07
	SAL	*overall*	1738.27	672.58
	AMPH	*1st*	4293.71	817.40
		4th	4982.59	1075.11
WKY	AMPH	*overall*	3029.07	943.41
	SAL	*overall*	859.39	570.06
	AMPH	*1st*	2598.40	965.07
		4th	3459.75	724.49

Means and standard deviations for all groups with significant differences in traveled distance. M = mean, SD = standard deviation, W = Wistar line, WKY = Wistar-Kyoto line, AMPH = amphetamine, SAL = saline.

NUMBER OF TRILLS, FM CALLS AND FLATS: DESCRIPTIVE DATA

Although statistical analysis of the number of trills, FM calls and flats was not conducted, we provide descriptive data for these ~~two~~ categories in Supplementary table S3 for additional information. All data is presented non-transformed.

Table S3

			M	*SD*
		Overall trills and flats		
W	AMPH	*trills*	38.33	58.74
		FM calls	602.54	318.54
		flats	262.25	212.62
	SAL	*trills*	0.04	0.20
		FM calls	5.58	9.56
		flats	2.83	6.88
	MORPH	*trills*	0.79	3.30
		FM calls	12.17	26.35
		flats	1.42	2.57
WKY	AMPH	*trills*	6.96	12.57
		FM calls	78.04	87.24
		flats	13.88	22.37
	SAL	*trills*	0.00	0.00
		FM calls	1.58	2.41
		flats	0.29	0.69
	MORPH	*trills*	0.00	0.00
		FM calls	0.58	1.18
		flats	0.08	0.28
		Trills and flats per each administration		
W	AMPH	*trills: 1st admin.*	37.50	69.34
		trills: 4th admin.	39.17	49.05

		FM calls: 1st admin.	445.42	279.43
		FM calls: 4th admin.	759.67	283.22
		flats: 1st admin.	159.92	136.38
		flats: 4th admin.	364.58	230.39
	SAL	*trills: 1st admin.*	0.00	0.00
		trills: 4th admin.	0.08	0.28
		FM calls: 1st admin.	2.83	3.07
		FM calls: 4th admin.	8.33	12.85
		flats: 1st admin.	0.83	2.59
		flats: 4th admin.	4.83	9.13
	MORPH	*trills: 1st admin.*	0.25	0.87
		trills: 4th admin.	1.33	4.62
		FM calls: 1st admin.	11.42	20.67
		FM calls: 4th admin.	12.92	31.98
		flats: 1st admin.	1.67	2.61
		flats: 4th admin.	1.17	2.62
WKY	AMPH	*trills: 1st admin.*	0.25	0.62
		trills: 4th admin.	13.67	15.23
		FM calls: 1st admin.	6.83	11.02
		FM calls: 4th admin.	149.25	68.76
		flats: 1st admin.	3.08	7.66
		flats: 4th admin.	24.67	27.09
	SAL	*trills: 1st admin.*	0.00	0.00
		trills: 4th admin.	0.00	0.00
		FM calls: 1st admin.	1.17	1.64
		FM calls: 4th admin.	2.00	3.02
		flats: 1st admin.	0.25	0.62
		flats: 4th admin.	0.33	0.78
	MORPH	*trills: 1st admin.*	0.00	0.00
		trills: 4th admin.	0.00	0.00
		FM calls: 1st	0.50	1.00

		admin.		
		FM calls: 4th admin.	0.67	1.37
		flats: 1st admin.	0.08	0.29
		flats: 4th admin.	0.08	0.29

Means and standard deviations for the number of trills and flats in all groups. M = mean, SD = standard deviation, W = Wistar line, WKY = Wistar-Kyoto line, AMPH = amphetamine, SAL = saline, MORPH = morphine, FM calls = frequency-modulated calls, admin. = administration.

CONDITIONED PLACE PREFERENCE: DESCRIPTIVE DATA

In the Supplementary table S4 we present the descriptive data for time spent in drug-paired vs. SAL-paired compartment during the CPP test (after 5 min., after 15 min., after 30 min.). Data for WKY25 in L compartment after 15 min. was missing, therefore WKY25 was excluded for that analysis. All data is log-transformed.

Table S4

			M	*SD*
		Time spent in compartment (0-5 min.)		
W	AMPH	*drug-paired*	4.44	0.23
		SAL-paired	4.12	0.64
	SAL	*drug-paired*	4.33	0.25
		SAL-paired	4.37	0.29
	MORPH	*drug-paired*	4.15	0.34
		SAL-paired	4.42	0.35
WKY	AMPH	*drug-paired*	4.15	0.34
		SAL-paired	4.18	0.30
	SAL	*drug-paired*	4.26	0.27
		SAL-paired	4.32	0.16
	MORPH	*drug-paired*	4.27	0.37

		SAL-paired	4.23	0.40
Time spent in compartment (0-15 min.)				
W	AMPH	*drug-paired*	5.51	0.26
		SAL-paired	5.39	0.31
	SAL	*drug-paired*	5.44	0.25
		SAL-paired	5.50	0.34
	MORPH	*drug-paired*	5.17	0.40
		SAL-paired	5.45	0.37
WKY	AMPH	*drug-paired*	5.32	0.50
		SAL-paired	5.28	0.27
	SAL	*drug-paired*	5.39	0.34
		SAL-paired	5.49	0.46
	MORPH	*drug-paired*	5.62	0.40
		SAL-paired	5.16	0.35
Time spent in compartment (0-30 min.)				
W	AMPH	*drug-paired*	5.93	0.42
		SAL-paired	6.07	0.43
	SAL	*drug-paired*	6.05	0.33
		SAL-paired	6.17	0.33
	MORPH	*drug-paired*	5.77	0.53
		SAL-paired	6.12	0.55
WKY	AMPH	*drug-paired*	5.75	0.68
		SAL-paired	5.92	0.78
	SAL	*drug-paired*	6.13	0.58
		SAL-paired	6.30	0.66
	MORPH	*drug-paired*	6.35	0.70
		SAL-paired	5.91	0.58

SUCROSE PREFERENCE: DESCRIPTIVE DATA

Supplementary table S5 presents descriptive data on significant differences between groups tested for sucrose consumption, normalized by weight. All data is presented non-transformed and expressed in proportions (grams of sucrose consumed / body weight).

Table S5

	M	SD
	SPT 1	
W	0.29	0.15
WKY	0.19	0.07
	SPT 2	
W	0.17	0.07
WKY	0.13	0.06

Means and standard deviations for all groups with significant differences in sucrose preference. M = mean, SD = standard deviation, SPT1 = first sucrose preference test, SPT2 = second sucrose preference test, W = Wistar line, WKY = Wistar-Kyoto line, AMPH = amphetamine, SAL = saline.

ANTICIPATORY 50-kHz CALLS AND LOCOMOTION

To further assess motivational outcomes, we measured anticipatory 50-kHz calls and locomotor activity prior to the fourth and final drug administration. All rats were placed in the drug-associated compartment 10 min. prior to the fourth administration. Anticipatory 50-kHz calls and locomotion were recorded, except for one animal, due to equipment malfunction. The data analyses followed the same pipeline as outlined in the Methods section. The descriptive data for significant differences is presented in the Supplementary table S5. Data on 50-kHz calls is log-transformed and the data on locomotor activity is non-transformed.

Table S5

	M	*SD*

Anticipatory 50-kHz calls (log(N))			
W	*overall*	3.57	1.25
WKY	*overall*	1.98	1.07
Anticipatory locomotor activity (cm)			
W	*overall*	3374.54	547.31
WKY	*overall*	2013.82	533.30

Means and standard deviations for all groups with significant differences for anticipatory 50-kHz calls and locomotor activity.

2 x 3 (Line x Treatment group) between-subjects ANOVAs on log-transformed number of 50-kHz calls revealed only a significant effect of line ($F(1,65) = 32.76$; $p < 0.0001$; $\omega^2 = 0.31$ with 95% CI [0.13, 0.49]). A similar main effect was observed for traveled distance ($F(1,66) = 111.50$; $p < 0.0001$; $\omega^2 = 0.61$ with 95% CI [0.44, 0.73]). Binary logistic regression examining the association between drug treatment and the probability of vocalizing trill or flat calls showed no significant predictors in either line. The results are illustrated in the Supplementary Figure 1 (Fig. S1).

Fig. S1

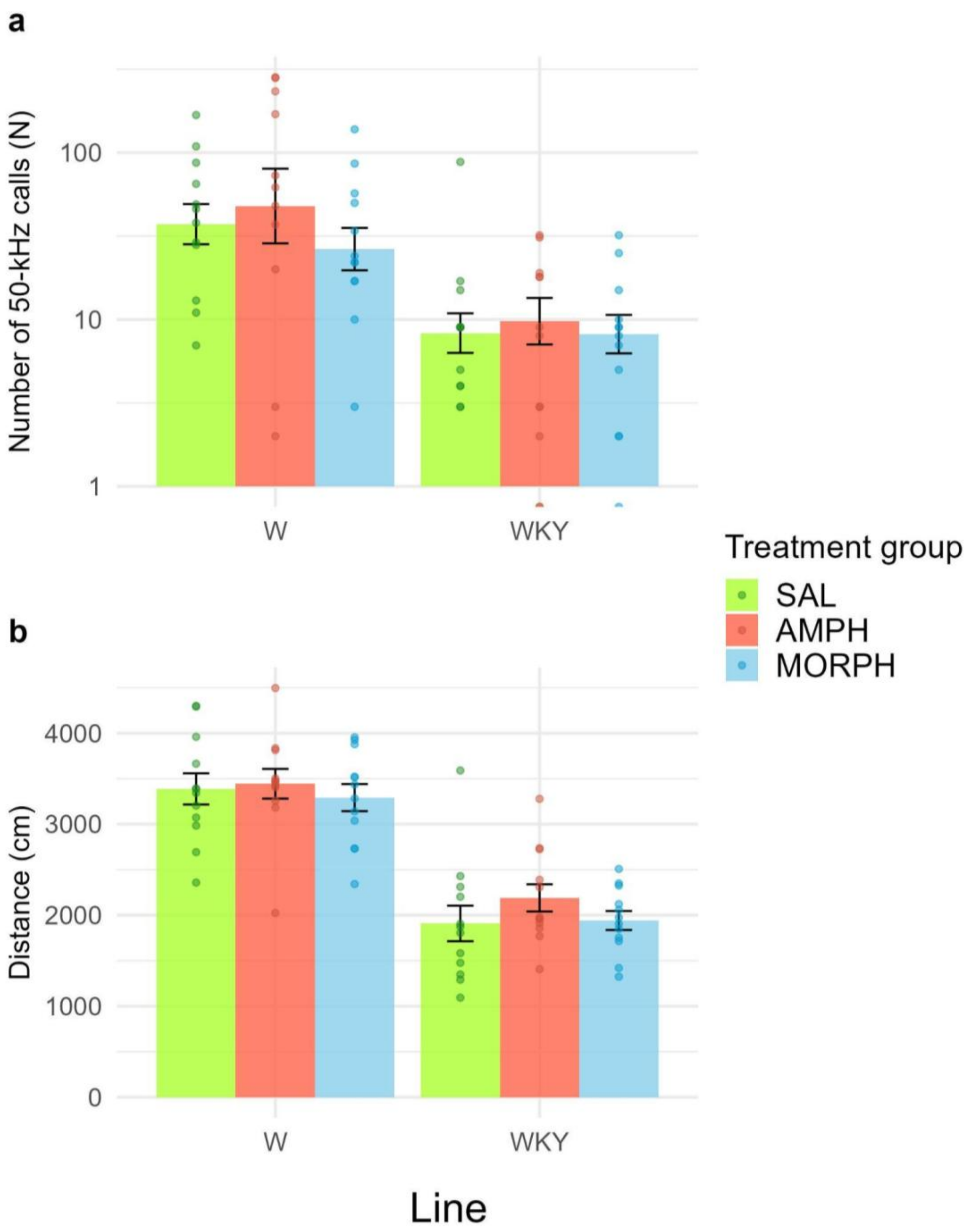

Anticipatory 50-kHz calls and locomotion in W and WKY before treatment with AMPH and MORPH. 10 min. prior to the fourth administration, rats were put in the drug-paired compartment for measurements of anticipatory 50-kHz calls and locomotor activity. Panel a describes the number of anticipatory 50-kHz calls (N) before SAL, AMPH, or MORPH in W and WKY rats. Panel b describes the distance traveled in anticipation (cm) before SAL, AMPH, or MORPH in W and WKY rats.
